# Stimulus-Responsive Afterglow Carbon Dots from Internal Mechanism to Potential Application

**DOI:** 10.3390/nano15231769

**Published:** 2025-11-25

**Authors:** Chongye Xia, Xingyu Gu, Xingwang Zhu, Yunfei Sun, Qijun Li, Jing Tan

**Affiliations:** 1 Institute of Technology for Carbon Neutralization, Yangzhou University, Yangzhou 225009, China; 2 College of Physical Science and Technology, Yangzhou University, Yangzhou 225009, China; 3 School of Electronic and Information Engineering, Suzhou University of Science and Technology, Suzhou 215009, China; 4 School of Mechanical Engineering, Yangzhou University, Yangzhou 225009, China

**Keywords:** carbon dots, afterglow, stimulus-responsive, sensing, information encryption

## Abstract

Stimulus-responsive afterglow materials refer to a class of substances whose afterglow characteristics alter under external stimuli, showing considerable potential for advanced applications in anti-counterfeiting, optoelectronic displays, chemical sensing, and bioimaging. Carbon dots (CDs), as an emerging category of afterglow materials, have garnered significant attention due to their stable photophysical and chemical properties, low toxicity, and tunable luminescent energy bands. In recent years, significant progress has been made in the development of stimulus-responsive afterglow CDs, underscoring the need for a systematic summary of this rapidly advancing field. This review summarizes recent advances in CD-based afterglow, encompassing luminescence mechanisms and synthesis strategies. A particular focus is placed on the types of stimulus-responsive afterglow behaviors in CDs, their influence on afterglow performance, and the underlying response mechanisms. The potential applications of these stimulus-responsive afterglow CDs in sensing and information encryption are also discussed in detail. Finally, current challenges and future prospects are outlined, aiming to guide the rational design and development of next-generation stimulus-responsive afterglow CDs.

## 1. Introduction

Stimulus-responsive afterglow materials refer to a class of substances whose photophysical and photochemical properties undergo changes upon exposure to external stimuli, such as light, heat, or mechanical force [1]. These materials hold great promise for applications in advanced anti-counterfeiting [2,3], optoelectronic displays [4], chemical sensing [5], and life sciences [6]. Compared to conventional fluorescent materials, afterglow materials exhibit prolonged luminescence lifetimes and enable multidimensional monitoring under external stimuli, offering unique advantages across various domains. However, most conventional room-temperature afterglow materials are inorganic compounds or organic complexes containing precious metals. Such materials often suffer from limitations including poor processability, high energy consumption during synthesis, and potential toxicity, which hinder their practical application [7]. In recent years, increasing attention has focused on metal-free, purely organic room-temperature afterglow materials. Researchers have achieved stimulus-responsive afterglow color changes in purely organic room-temperature afterglow materials by structural modification of the luminescent core via adjustments in molecular structure, crystalline phase, packing arrangement, or conformation through methods, such as grinding [8,9,10], thermal annealing [11], or solvent atmosphere induction [12,13]. Nevertheless, these systems often require additional treatments such as heating or recrystallisation to restore luminescence, exhibiting an absence of reversible response to external stimuli. Moreover, their intricate synthesis strategies and insufficient photostability restrict their practical applications. Therefore, there is an urgent need to develop a new generation of stimulus-responsive afterglow materials, which feature simple preparation processes, environmental friendliness and non-toxicity, excellent light stability and cost-effectiveness [14].

Carbon dots (CDs) are zero-dimensional carbon-based nanomaterials, characterized by a sp^2^/sp^3^ carbon core and an outer layer of functional groups or organic molecules, typically with particle sizes below 10 nm [15]. Compared to conventional afterglow materials, CDs offer distinct advantages such as facile synthesis, stable photophysical and photochemical properties, low toxicity, and tunable emission bands, making them highly promising candidates for room-temperature afterglow applications [16,17,18,19,20,21,22,23,24]. However, achieving high-performance afterglow emission in CDs at room temperature remains challenging due to weak spin–orbit coupling and rapid non-radiative decay. To address these limitations, two primary strategies have been developed. On one hand, On one hand, efforts focus on enhancing the intersystem crossing efficiency between singlet and triplet states, such as heteroatom doping (N [25,26,27,28], O [29,30,31,32], P [33,34]). On the other hand, strategies aim to suppress non-radiative transitions and block the quenching effect of oxygen [35,36,37,38]. These objectives can be accomplished either by embedding CDs within a matrix material (e.g., polyvinyl alcohol [39,40,41,42,43], silica [44,45,46,47], or boric acid [48,49,50,51]) or constructing a polymer-like shell on their surface to restrict molecular vibration and rotation.

After nearly a decade of development, room-temperature afterglow CDs have achieved remarkable performance, with reported afterglow quantum yields of up to 50% [52], full-spectrum spanning ultraviolet to near-infrared light [53], and long-lifetime with afterglow visible for several hours [54]. These properties are now competitive with those of conventional afterglow materials. However, the inherent incompatibility between matrix encapsulation and external stimulus response has hindered the development of stimulated-response afterglow CDs. In 2016, our group first reported oxygen-responsive phosphorescence by embedding CDs in a polyurethane matrix, where photosensitization was employed to consume oxygen and induce light-triggered phosphorescence enhancement [55]. Subsequently, Yang and co-workers demonstrated pH-responsive phosphorescence intensity modulation by depositing N,P-doped CDs onto paper substrates [56]. Lin’s team achieved the conversion of fluorescent-emitting CDs into ultra-long room-temperature phosphorescence (RTP) through external heating stimulation [34]. Our team further enhanced phosphorescence by leveraging water molecules to form hydrogen-bonding networks between CDs and the surrounding matrix [57]. Despite these advances, most CDs exhibit only intensity variations in their afterglow emission due to the presence of a single afterglow center, making visual discrimination difficult and often requiring instrumental detection. In 2021, our group proposed a novel approach to achieve time-dependent phosphorescence colors in CDs by creating dual phosphorescent centers [58]. More recently, we developed a surface ionization engineering approach to achieve tunable dynamic phosphorescence color on paper. Under strong alkaline conditions, ionization of phenolic hydroxyl groups on the CD surface enhances inter-system crossing efficiency, activating surface-state associated blue phosphorescence. Combined with carbon core-state associated yellow-green phosphorescence, this system exhibits excitation- and time-dependent color switching from yellow-green to blue, providing a versatile platform for advanced optical applications [59].

Over the past few years, considerable progress has been made in the field of stimulus-responsive afterglow CDs. This review systematically summarizes the emission mechanisms and synthetic methods of such materials. Following the introduction, Section 2 elucidates the fundamental mechanisms of afterglow in CDs and outlines the main preparation strategies—namely matrix-assisted and self-protective methods—for constructing stable afterglow centers. Section 3 provides a detailed discussion on various stimulus-responsive types, such as temperature, moisture, pH, and light, along with their underlying influence mechanisms on afterglow performance. Section 4 explores the potential applications of these stimulus-responsive afterglow CDs in sensing and information encryption/anti-counterfeiting. Finally, Section 5 concludes with a summary and insightful perspectives on future research directions.

## 2. Mechanism and Preparation Methods for Afterglow CDs

**Figure 1:**
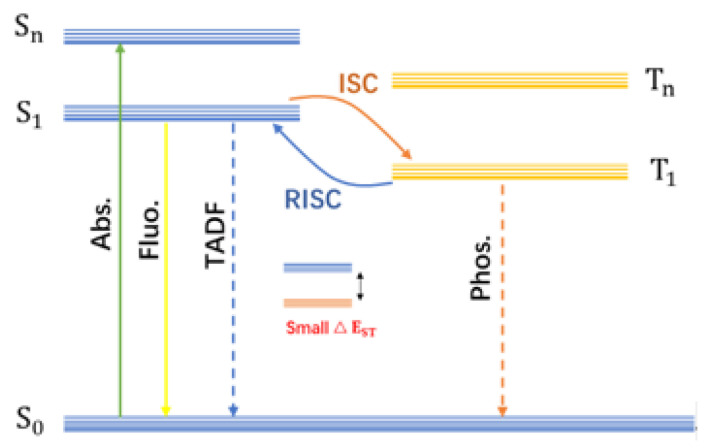
Jablonski energy level diagram.

The luminescence mechanism of CDs is complex, yet it is primarily attributed to two dominant contributing centers: the carbon core state and the surface state. The carbon core typically consists of sp^2^-hybridized carbon, which may be interspersed with sp^3^-hybridized carbons. The emission wavelength can be engineered by controlling the size of the sp^2^ domains—enlarging these domains causes a red shift in the emission. Furthermore, the surfaces of CDs are typically decorated with abundant functional groups (e.g., carboxyl, hydroxyl, amino) and contain numerous defects, such as broken chemical bonds or dangling bonds. These moieties create localized electronic states within the energy gap, known as “surface states.” Emission originating from these surface states typically exhibits strong excitation wavelength dependence and is highly sensitive to the local environment, including pH, ionic strength, and solvent polarity. Afterglow represents a specific category of photoluminescence, including phosphorescence and delayed fluorescence (DF) [60,61,62,63]. As show in Figure 1, upon irradiation with light of a specific wavelength, CDs absorbs photon energy, and electrons transition from the ground state (S_0_) to the excited singlet state (S_1_). In this state, the electron spin directions are opposite (↑↓), consistent with the Pauli exclusion principle. The radiative transition of electrons from S_1_ back to S_0_ results in fluorescence emission, which typically exhibits a nanosecond-scale lifetime. Alternatively, electrons in the S_1_ state may undergo intersystem crossing (ISC) to populate a triplet excited state (T_1_). The subsequent radiative transition from T_1_ to S_0_ yields phosphorescence. Owing to spin confinement (↑↑), the radiative transition rate from the triplet state is significantly slower, endowing phosphorescence with a characteristically long lifetime that can range from microseconds to hours.

When the energy gap between S_1_ and T_1_ is sufficiently small, the electrons of T_1_ can return to S_1_ through anti-intersystem crossing (RISC) and then radiate the transition in the form of fluorescence, which is called delayed fluorescence. DF is generally classified into three types: E-type DF or called thermally activated delayed fluorescence (TADF, emission arising from the S1 that populated by a RISC from T_1_), P-type delayed fluorescence (triplet−triplet annihilation, TTA-DF), and recombination fluorescence (recombination of radical ions or opposite charges). Among them, TADF has been the most widely reported due to its promising applications in optoelectronic devices. Based on their distinct emission mechanisms, TADF and phosphorescence can generally be distinguished by comparing their steady-state afterglow spectra at room temperature and low temperature. Specifically, at low temperatures (e.g., 77 K), molecular thermal motion is suppressed, and the ISC process is hindered, leading to a significant decrease or even disappearance in the efficiency of TADF. In contrast, phosphorescence originates from the direct radiative transition of triplet excitons and is less affected by temperature; thus, it maintains relatively strong emission intensity even at low temperatures. By analyzing the changes in intensity and decay behavior of the afterglow spectra under high and low temperature conditions, the two types of emission can be effectively distinguished. Although its spectral profile is identical to that of prompt fluorescence, DF exhibits a substantially longer lifetime, typically in the microsecond to second range. From the perspective of the energy level diagram (Figure 1), achieving efficient phosphorescence or DF emission crucially depends on both the population of triplet excitons and the suppression of non-radiative decay pathways. This entails the establishment and stabilization of robust afterglow luminescence centers within the CDs.

### 2.1. Construction of the Afterglow Luminescence Center

According to the El-Sayed rule, the spin–orbit coupling values are typically low for transitions between states of similar electronic configurations, specifically from ^1^(π, π*) S_1_ to ^3^(π, π*) T_n_ or from ^1^(n, π*) S_1_ to ^3^(n, π*) T_n_, resulting in weak ISC capability. In contrast, SOC is significantly enhanced when the electron configuration changes between different types of states, such as from ^1^(π, π*) S_1_ to ^3^ (n, π*) T_n_ or from ^1^(n, π*) S_1_ to ^3^(π, π*) T_n_, thereby facilitating efficient ISC [64]. Owing to the unique sp^2^ structure of the carbon core, CDs exhibit abundant (π, π*) transitions. Introducing heteroatoms bearing lone electron pairs (e.g., N, O, P) into the carbon framework promotes the formation of high-density (n, π*) electronic configurations within the CDs sp^2^ conjugated system (Table 1). This enhances the spin–orbit coupling between S_1_ and T_1_ states and reduces the energy range difference (ΔE_ST_) between the excited S_1_ and T_1_, effectively promoting the triplet exciton generation. Through TD-DFT calculations on CD models before and after urea treatment, Song’s team found that introducing electron-rich functional groups (–NH_2_, C=O, C≡N) significantly reduced ΔEST from 0.248 eV to 0.006 eV. These functional groups introduce (n, π*) excited state configurations into the π-π* conjugation system of the CDs. This change is the fundamental reason for the increase in the spin–orbit coupling constant (ξ) from 0.03 cm^−1^ to 3.35 cm^−1^, confirming that heteroatom doping enhances ISC by altering the nature of the excited state. According to El-Sayed’s rule, electronic configuration changes between (π, π*) and (n, π*) can greatly enhance spin–orbit coupling, thereby efficiently driving the ISC process [52].

**Figure 2:**
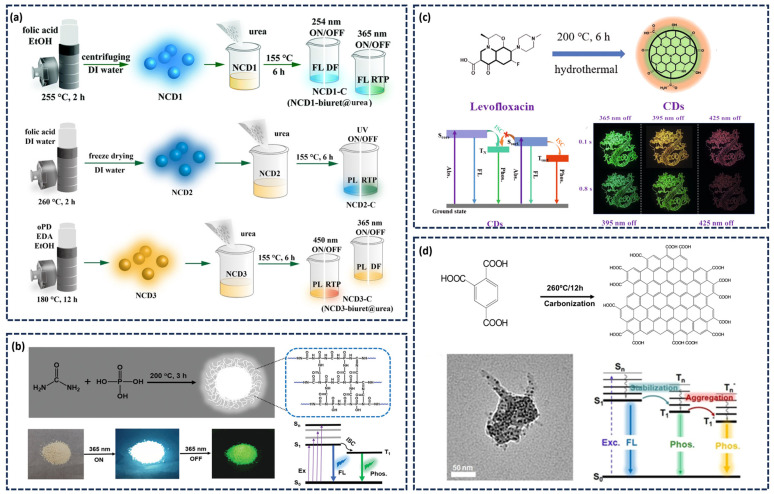
(**a**) Schematic illustration of the preparation process of CDs [65]; (**b**) Preparation and luminescence mechanism of a single-phase white-light-emitting CDs and the diagram of the luminescence process [66]; (**c**) Synthesis process, luminescence mechanism, and demonstration of multicolor dynamic phosphorescence from CD-based inks under UV excitation [58]; (**d**) Schematic diagram of the synthesis and phosphorescence emission of TA-CDs [67].

In 2013, the phosphorescence phenomenon of CDs was first reported, and its phosphorescence was mainly attributed to the aromatic carbonyl groups in CDs [39]. The strong spin–orbit coupling effect of aromatic carbonyl compounds promotes the generation of CDs triplet excitons. In 2016, our research group proposed that nitrogen doping could enhance the ISC efficiency in CDs. We systematically investigated the influence of the nitrogen doping form in CDs on its band structure, triplet excited state properties, as well as the phosphorescence emission intensity and lifetime. For the first time, we demonstrated that C=N groups can serve as effective phosphorescence emission center of CDs [55]. Lin’s group precisely tailored the energy level structure of CDs by altering the nitrogen doping amount and the type of functional groups [65], preparing three distinct N-CDs that exhibited wavelength-selective excitation of polychromatic RTP spanning blue, green to red (Figure 2a). Boron, located adjacent to nitrogen in the periodic table and sharing similar characteristics, has also been employed for doping. Feng and colleagues synthesized a series of boron-doped CDs using citric acid and boric acid as precursors. The electron-deficient nature of boron atoms induces polarization within the π-electron system of CDs, reducing the S_1_ and T_1_ energy gap and promoting ISC, thereby endowing the resulting CDs with an exceptionally long RTP lifetime [51]. Phosphorus, as another group VA element possessing lone-pair electrons, also facilitates n-π* transition and enhances ISC. Wang’s group synthesized a single-phase white light CDs material with a phosphorescent quantum yield as high as 23% via a one-step hydrothermal treatment of urea and phosphoric acid [66]. Studies have shown that the high concentration co-doping of N and P elements reduces the energy gap between S_1_ and T_1_, thereby enhancing the ISC process and promoting efficient phosphorescence emission from CDs (Figure 2b). The co-doping of heteroatoms can also establish multiple phosphorescent luminescence centers in CDs. As shown in Figure 2c, for the first time, our group synthesized CDs featuring dual phosphorescent luminescent centers (nitrogen heterocyclic and aromatic carbonyl) via a hydrothermal method using levofloxacin as the precursor [58]. Nitrogen heterocycles were primarily inherited from the precursor, while aromatic carbonyl groups formed during the hydrothermal process. The two phosphorescent centers are simultaneously activated, yielding fast-decaying red phosphorescence and slow-decaying green phosphorescence. By leveraging the distinct emission wavelengths, intensity, and lifetime of these two independent luminescent centers, the CDs were printed onto paper to obtain time-responsive color-changing phosphorescence.

Beyond the introduction of lone-pair electrons into CDs, aggregation-induced energy level splitting represents another effective strategy for constructing afterglow centers in CDs. When chromophores aggregate from monomers into aggregates, strong electronic interactions lead to exciton orbital overlap and consequent energy level splitting. This splitting can create additional ISC channels, and increase the luminescence band gap and luminescence efficiency and afterglow in aggregates. Lin’s team prepared trimellitic acid (TA) as the raw material and prepared TA-CDs with long-lived yellow phosphorescence emission through the hydrothermal method. A tight aggregate is formed between TA and CDs through a strong π-π stacking interaction [67]. They pointed out that this aggregation give rise to an additional triplet excited state (T_n_*) with a lower energy level (Figure 2d), resulting in longer-wavelength phosphorescence emission observable as a yellow afterglow by the naked eye.

### 2.2. Stability of the Afterglow Emission Center

The long-lived triplet excitons of CDs are highly susceptible to deactivation through non-radiative pathways, including thermodynamic vibration and quenching by environmental factors such as oxygen. Establishing a rigid microenvironment to minimize excited-state energy loss represents an effective strategy for achieving and enhancing afterglow luminescence efficiency. Two primary approaches have been developed, including the matrix-assisted and self-protection methods.

#### 2.2.1. Matrix-Assisted Method

This approach relies on embedding CDs within a supporting matrix to stabilize their afterglow emission. The strong covalent or hydrogen bonding interactions between the matrix and CDs, combined with the resulting spatial confinement and oxygen barrier effects, collectively suppress molecular vibrations, mitigate non-radiative transitions, and prevent oxygen-induced quenching. This constitutes the predominant strategy for stabilizing the afterglow luminescence center. Matrices are broadly categorized into organic and inorganic materials (e.g., silica, boric acid).

**Figure 3:**
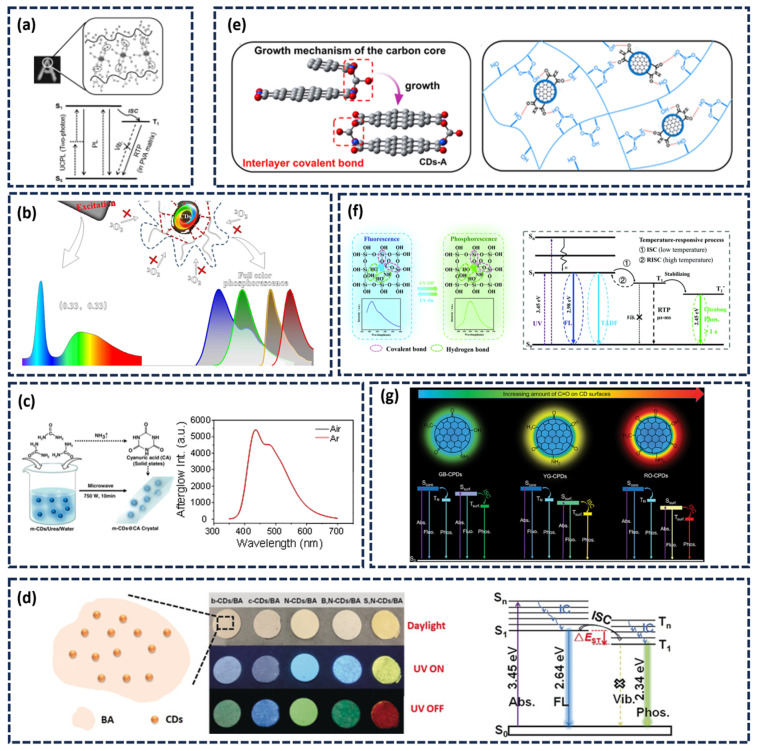
(**a**) Schematic representation of the triple mode emission of CDs-PVA [40]; (**b**) Phosphorescent emission spectra of CDs [77]; (**c**) Schematic illustration of the preparation process of m-CDs@CA and afterglow emission spectra of m-CDs@CA composite under air and argon atmospheres [54]; (**d**) Digital photographs of CDs/BA composites and corresponding energy level diagrams of CDs@BA [69]; (**e**) Proposed growth mechanism of the carbon core and schematic illustration of the multiple hydrogen bonding between CDs and matrix [70]; (**f**) Chemical interaction mechanism between CDs and the Si–O network, accompanied by the energy level diagram of CDs@SiO_2_ composites [46]; (**g**) Model for dynamic full-color TP of these PDs [76].

Commonly used organic materials include polyvinyl alcohol (PVA) and polyurethane (PU). Their hydroxyl-rich chains can form extensive hydrogen bonds with functional groups on the CDs, such as C=O and C=N. This effectively immobilizes the phosphorescent moieties, reducing energy dissipation caused by molecular vibration or rotation, thereby enhancing afterglow efficiency [38]. Furthermore, the rigidity effect and oxygen isolation effect of the above-mentioned matrix can suppress non-radiative attenuation pathways, maximizing afterglow emission efficiency. For instance, Jiang’s group utilized the hydrogen bond interaction between PVA molecules and CDs to suppress the rotation and vibration of the CDs radiation center and the non-radiative transitions of the triplet excitons to stably excite the triplet state, achieving long-lived RTP emission of CDs (Figure 3a) [40]. Our research group successfully achieved RTP by incorporating CDs into a PU matrix, attributing the success to the combined effects of the polymer’s inherent rigidity and the formed hydrogen-bonding network [55]. Similarly, Wang and colleagues dispersed CDs into PAM, producing a material capable of emitting four phosphorescent colors. They concluded that the hydrogen bonds formed between the PAM matrix and CD increase the environmental rigidity, suppress the vibration, rotation and collision of the luminescent clusters (C=O), and isolate oxygen. This comprehensive suppression of non-radiative transitions significantly promotes phosphorescence generation in the composite material (Figure 3b) [77].

In addition to organic polymers, small organic molecules can also serve as effective matrices to produce afterglow. Jiang’s team developed CDs with a phosphorescent lifespan of several hours through simple microwave heat treatment of CDs and urea [54]. During the heating process, urea undergoes conversion to cyanuric acid (CA), forming a highly crystalline matrix. The hydrogen bonds in the CA crystals also enhance the rigidity of the matrix, while the crystalline structure restricts molecular motion and suppresses non-radiative transitions (Figure 3c). Moreover, the CA crystal lattice effectively shields the CDs from oxygen and moisture quenching. This protection was confirmed by the nearly identical afterglow intensities observed for CDs@CA powders under both air and argon atmospheres.

Inorganic materials provide an alternative stabilization approach, leveraging their inherent rigidity and strong confinement effects to suppress non-radiative transitions and isolate oxygen and water vapor. Common inorganic materials mainly include boric acid, silicon dioxide, aluminum oxide [68]. Li’s research team developed a strategy involving the heat treatment of boric acid (BA) and CDs. During the process, covalent bonds were formed on the surface of the CDs. Meanwhile, by taking advantage of the strong hardening effect of the glassy state of BA, the CDs triplet excitons were effectively fixed, and the non-radiative transitions were suppressed. (Figure 3d). The resulting material achieved an extended phosphorescent lifetime of 1.6 s [69].

Li and his team members embedded CDs into a rigid polyboronic acid (pBA) matrix through pyrolysis [70]. The resulting composite demonstrated exceptional thermal stability, an extremely long lifespan, and multi-color phosphorescence, maintaining visible phosphorescence for 5 s even at 170 °C. The pBA matrix, with its decomposition temperature exceeding 200 °C and glass transition temperature of 140 °C, forms a cross-linked network that effectively suppresses molecular thermal motion. Additionally, the abundant surface hydroxyl groups of pBA create an extensive hydrogen-bonding network with the CDs (Figure 3e), further restricting molecular vibration and rotation to minimize energy loss.

Certain inorganic matrices further enhance afterglow through their defect structures, which can form trap energy levels that stabilize triplet excitons. Liu’s group synthesized CDs@SiO_2_ composite nanoparticles via a sol–gel method, obtaining materials exhibiting long-afterglow emission with lifetimes up to 1.76 s in both solid and liquid states [46]. The composite demonstrates dual emission centers for phosphorescence and delayed fluorescence. Thermoluminescence analysis revealed defect trap levels (STrap) within the SiO_2_ matrix that function as electron reservoirs, capable of capturing, storing, and gradually releasing excited-state electrons to both T_1_ and S_1_ levels, thereby promoting concurrent phosphorescence and delayed fluorescence emission (Figure 3f). Subsequently, they introduced CDs into the Al_2_O_3_ matrix [68]. The spatial confinement of Al_2_O_3_, combined with covalent and hydrogen bonding interactions, facilitates efficient intersystem crossing from S_1_ to T_1_. Furthermore, they demonstrated that multi-color afterglow emission could be achieved by adjusting the calcination temperature to regulate the surface functional groups and conjugation degree of the CDs. Shan’ team obtained an organic microrod (OMR) by heating benzoic acid and melamine, which exhibited strong phosphorescence in aqueous solution. Theoretical calculations suggested that the observed phosphorescence in water resulted from the formation of a hydrogen-bond network between water molecules and the OMR molecules. This hydrogen-bond network enhances the overall rigidity of the system, reduces the energy gap between the S_1_ and T_1_ excited states, and thereby enhances phosphorescence [71].

#### 2.2.2. Matrix-Free (Self-Protected) Afterglow CDs

Matrix-free RTP CDs is fundamentally derived from the “agglomeration-induced luminescence” mechanism. The highly cross-linked carbonized polymer structure formed on the CD surface functions similarly to an external matrix, stabilizing triplet excitons and facilitating intrinsic RTP emission [72,73,78]. Yang’s research group successfully prepared a series of self-protected RTP CDs via a one-step hydrothermal cross-linking polymerization method [74]. The research revealed that the cross-linking polymerization effect restricted the vibration of surface functional groups on the CDs, thereby activating phosphorescence emission. Zheng’s team further investigated the influence of polymer structure on the phosphorescence of both self-protected and matrix-assisted CDs, highlighting that a densely cross-linked internal polymer network can immobilize the phosphorescent centers. This rigid internal structure provides a stable self-confinement effect that induces and sustains phosphorescence [38]. Cross-linkable polymers or functional small molecules are widely employed in synthesizing such self-protective systems, typically exhibiting pronounced polymerization characteristics and a highly cross-linked, polymer-like architecture. Generally, a higher degree of cross-linking in CDs leads to more tightly confined afterglow centers and correspondingly higher phosphorescence efficiency. Feng’s group prepared fluorine-nitrogen doped CDs with self-protective RTP through a one-step solvothermal method [75]. The synergistic effect of hydrogen bonds and the steric shielding provided by C–F bonds within the system collectively reduce oxygen-induced phosphorescence quenching at room temperature. More recently, Ding’s team fabricated chromic tunable RTP self-protective CDs by heat-treating a mixture of urea, 1,8-naphthalendiformimide and quinacridone (Figure 3g) [76]. They simulated model units with different C=O contents and different aggregation states (monomer → dimer → trimer) through DFT calculations. The results showed that the surface state energy levels underwent a redshift as the structure changed. This directly explains the observed full-color, time-dependent phosphorescence phenomenon in experiments. This intrinsic afterglow emission is primarily attributed to the extensive hydrogen-bonding network formed during the CDs aggregation process. The increase in both the surface oxygen content and the aggregation degree induces a redshift in the afterglow emission and results in a broad emission peak. Tao et al. compared the phosphorescence properties of a single luminescent unit with those of a cross-linked coupled unit (dimer). The phosphorescence wavelength (494 nm) of the calculated dimer is in perfect agreement with the experiment. It was hypothesized that the cross-linked structure restricts intramolecular rotation, provides a rigid environment, and stabilizes triplet excitons. This theoretically confirms the Cross-Linking Enhanced Emission (CEE) effect as the core mechanism for RTP generation in polymer CDs [18].

## 3. Stimulus-Responsive Types and Their Impact on Afterglow Performance

Although the luminescence mechanism of CDs afterglow has been progressively elucidated, achieving dynamic modulation of afterglow properties through external stimuli remains a pivotal challenge. The core feature of stimulus-responsive afterglow CDs lies in their dynamic optical properties, including intensity, color, and lifetime, in response to external stimuli. Based on the controllable characteristics of its luminescent mechanism, external stimuli can intervene in the exciton relaxation pathways or reconstruct the energy level structure, endowing stimulus-responsive afterglow CDs with a unique bidirectional interaction capability characterized by “stimulus-response.” Common stimulus types include temperature, humidity, pH, light, etc. (Table 2).

### 3.1. Temperature-Responsive Afterglow

Temperature modulates afterglow intensity primarily by altering the ISC efficiency. At lower temperatures, reduced molecular vibration enhances ISC from S_1_ to T_1_, increasing the triplet exciton population and thereby intensifying afterglow emission. Conversely, elevated temperatures amplify molecular thermal motion, promoting non-radiative energy dissipation pathways such as internal conversion (IC), reducing the efficiency of ISC. From the perspective of emission mechanism, phosphorescence arises from the radiative transition from T to S. At high temperatures, molecules in the T_1_ state are more likely to return to the S_0_ via non-radiative pathways, such as vibrational coupling and collision, resulting in phosphorescence quenching. This process can be described by the Arrhenius Equation:

knr=Ae−EaRT

Here, $k_{nr}$ denotes the non-radiative decay rate, and $E_{a}$ represents the activation energy. At high temperatures, the $k_{nr}$ of T_1_ increases, the proportion of radiative transition decreases, and the phosphorescence is quenched.

**Figure 4:**
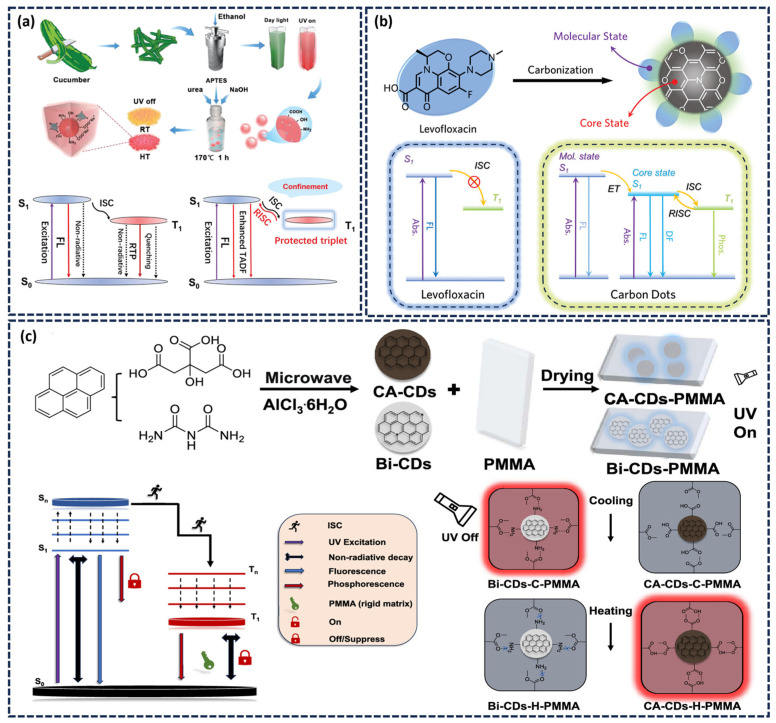
(**a**) Design strategy for uCDs@SiO_2_/NaOH composites and the mechanism of their thermally enhanced DF emission [79]; (**b**) Preparation process of thermoresponsive CDs and corresponding Jablonski diagram of the luminescence pathways in levofloxacin and CDs [80]; (**c**) Synthesis route and operating mechanism of temperature-responsive stimulated red phosphorescent materials, along with the proposed phosphorescence mechanism in CDs-PMMA composites [81].

By comparing the afterglow intensity at high and low temperatures, it can determine whether the afterglow is phosphorescence or thermally delayed fluorescence. As the temperature rises, the thermal motion of molecules intensifies, the thermal activation energy increases, and the RISC rate of triplet excitons significantly accelerates. More triplet excitons return to S_1_, which is manifested macroscopically as an enhanced intensity of DF. Shan’s research team introduced CDs into ionic crystal networks and obtained thermally enhanced DF materials [79]. As shown in Figure 4a, covalent/hydrogen bonds between CDs and matrix suppress triplet non-radiative transitions to promote phosphorescence, and rapid RISC is crucial for DF generation. Recently, our team achieved thermochromic CDs-based afterglow materials by melting BA and levofloxacin at high temperatures (Figure 4b). At low temperature (193 K), insufficient thermal activation energy favors the ISC process, leading to exciton transfer from the S_1_ to the T_1_, resulting in yellow phosphorescent emission centered at 560 nm. As the temperature rises, the increase in thermal activation energy improves RISC efficiency, enabling the excitons reverse transition from T_1_ to S_1_ by absorbing thermal energy. Therefore, at high temperatures (373 K), TADF becomes the dominant emission mode, appearing as a blue afterglow. Owing to different proportions of DF and phosphorescence at various temperatures, the afterglow color shifts from yellow at low temperature to blue at high temperature, enabling wide-range thermochromic afterglow regulation [80].

Furthermore, temperature can modulate the phosphorescence intensity by altering the interactions between CDs and the surrounding matrix, thereby influencing the rate of exciton radiative transitions. Kang and colleagues demonstrated this effect by constructing a temperature-responsive red phosphorescent composite [81]. They prepared Bi-CDs and CA-CDs, and embedded them in the PMMA matrix. At high temperatures (70 °C), PMMA undergoes pyrolysis, releasing numerous hydroxyl groups, which form stable hydrogen bonds with the carboxyl groups on the surface of CA-CDs, promoting the ISC process and enhancing phosphorescence emission. In contrast, the hydrogen bonds formed with amino groups on the surface of Bi-CDs are relatively weak at high temperatures, resulting in increased molecular motion and non-radiative transformation rate, followed by phosphorescence quenching. The hydrogen bond behavior reverses upon cooling to room temperature. Bi-CDs reestablish hydrogen bonds with P the PMMA matrix, restoring phosphorescence emission. Conversely, the hydrogen bonds between CA-CDs and PMMA weaken or break upon cooling, causing the phosphorescence to disappear (Figure 4c).

### 3.2. Moisture-Responsive Afterglow

The influence of moisture on afterglow is multi-faceted and involves competing mechanisms. Water molecules possess high-energy O-H stretching vibrations that can couple with the T_1_ of the phosphor through vibronic interactions, promoting non-radiative transitions through vibration-electron coupling and dissipating energy in the form of heat, a process analogous to IC or ISC that ultimately reduces phosphorescence efficiency. Additionally, moisture often coexists with dissolved oxygen, particularly in liquid water or high-humidity environments, where oxygen can further quench triplet excitons and suppress phosphorescence. Nie et al. systematically investigated the effect of humidity on the afterglow lifetime of CDs and successfully developed N-CD-based test paper for RH monitoring.

**Figure 5:**
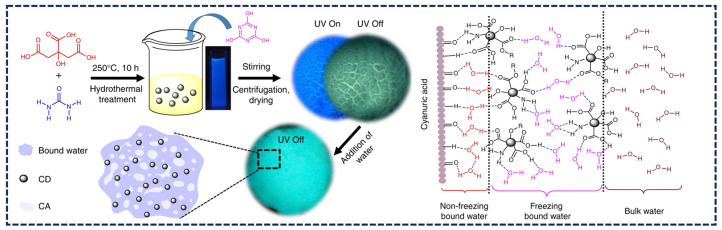
The preparation of CDs and CD-CA systems and the interaction between CDs, CA particles and water molecules [57].

In specific systems, water molecules can significantly enhance the rigidity of the system and suppress non-radiative transition processes by forming stable hydrogen bonds or other strong interaction forces with the matrix, thereby effectively promoting the radiative attenuation of triplet excitons. Our team utilized the bridging hydrogen bond network formed by water molecules between CDs and CA (Figure 5). The hydrogen bond network effectively hardened the C=O bond, enhancing the rigidity of the system [57]. Consequently, rather than causing phosphorescence quenching, the presence of water molecules substantially increased phosphorescence intensity. Furthermore, water molecules can also modulate excited-state energy level structures and alter molecular orbital coupling state, leading to an increase in phosphorescence intensity and a tunable afterglow color. Han’s group developed TPA-CDs/Si materials with unique reversible water-responsive color-changing afterglow properties using terephthalic acid as the carbon source and silicon dioxide as the silicon source. Through water-induced CDs self-assembly, they achieved phosphorescence color switching from blue to green [92]. This color-changing mechanism is mainly attributed to the fact that in the dry state, TPA-CDs/Si emits blue light due to the stable T_1_ of the Si-C bond. Upon hydration, water molecules form an extensive hydrogen-bonding network with oxygen-containing functional groups on the CD surface, which preferentially stabilizes the green RTP emission center while suppressing the blue DF emission, thereby causing the luminescence color to change from blue to green. This hydration-induced color change is fully reversible. After dehydration, the hydrogen bond network is disrupted and the blue light is restored. It is worth noting that the blue afterglow could be rapidly recovered by heating the written characters at 600 °C for merely 10 s.

### 3.3. pH-Responsive Afterglow

**Figure 6:**
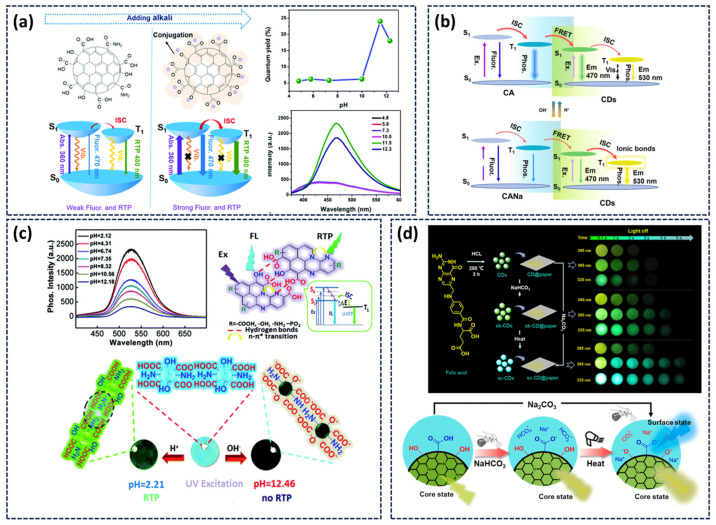
(**a**) The possible mechanism of enhanced phosphorescence, phosphorescence spectra and quantum yields of CD-based composites [82]; (**b**) pH-Dependent phosphorescence spectra of CDs and schematic illustration of their pH sensing mechanism [83]; (**c**) Mechanism of pH-stimulated tunable afterglow emission through selective activation of different exciton pathway [84]; (**d**) Schematic representation of the phosphorescence behavior and underlying mechanism in CDs before and after alkaline treatment and heating [59].

The pH responsiveness of CDs afterglow primarily arises from the sensitive response of their surface functional groups and the interactions with the surrounding matrix to environmental pH. CDs typically possess surfaces rich in functional groups such as carboxyl (-COOH) and hydroxyl (-OH), which undergo protonation or deprotonation under varying pH conditions. This alters the surface charge state of the CDs and consequently modulates their afterglow properties. Our team demonstrated this effect by embedding CDs into a CA matrix. Under alkaline conditions, the deprotonation of surface carboxyl groups enhanced the interaction between CDs and CA while reducing surface defects. (Figure 6a) This suppression of non-radiative transitions for triplet excitons resulted in a more than threefold enhancement of phosphorescence intensity, marking the first report of base-induced phosphorescence enhancement in CDs [82]. Yang’s research group synthesized CDs functionalized with abundant phosphate groups. When coated onto paper, these CDs exhibited green phosphorescence under acidic conditions, with intensity gradually diminishing as pH increased [83]. This quenching was attributed to the deprotonation of the phosphate group in an alkaline environment, which disrupted the inter-CD hydrogen-bonding network. The consequent loss of rigidity reduced triplet exciton stability, decreased ISC efficiency, and enhanced non-radiative decay pathways, ultimately leading to phosphorescence attenuation (Figure 6b).

In addition to the variation in pH-responsive afterglow intensity, we also achieved the regulation of the afterglow color of the system by alkali-induced selective activation of different exciton transfer channels in CD@CA composites [84]. After alkaline treatment, CD@CA exhibited a distinct and reversible afterglow color shift from cyan to yellow. System analysis revealed two competing exciton pathways within the system: the inherent phosphorescence emission state of CDs and the energy resonance transfer emission state between CDs and CA (Figure 6c). The bluish afterglow is derived from the energy resonance transfer of CA and CDs, and the yellow phosphorescence stems from the inherent emission center of CDs. Alkali introduction strengthens the ionic bonding between CDs and CA, which suppresses non-radiative transitions and favors the intrinsic emission pathway, resulting in the yellow afterglow. Furthermore, CD@CA composites exhibit good light stability. It can be activated to maintain 90% of the afterglow intensity when placed in a bare environment for more than 30 days.

Furthermore, our research group realized multi-mode dynamic regulation of the phosphorescence color in a single CDs through strong alkali-induced ionization of surface phenolic hydroxyl groups [59]. This treatment activates time- and excitation-dependent afterglow color changes from yellow-green to blue. The phenolic hydroxyl ionization can increase the ISC rate, passivate surface defects, reduce non-radiative attenuation, and induce surface-state blue phosphorescence. Meanwhile, the nitrogen-doped carbon core maintains its intrinsic yellow-green phosphorescence due to its stable triplet energy level structure. The coexistence of these two phosphorescent centers enables dynamic color modulation from yellow-green to blue, achieved through their distinct phosphorescence decay kinetics (blue emission decays slower than yellow-green). As illustrated in Figure 6d, this dynamic behavior can be thermally controlled using a weak base. Upon heating, NaHCO_3_ decomposes into strong base Na_2_CO_3_, which subsequently induces phenolic hydroxyl ionization and activates the dynamic color-changing phosphorescence.

### 3.4. Light-Responsive Afterglow

**Figure 7:**
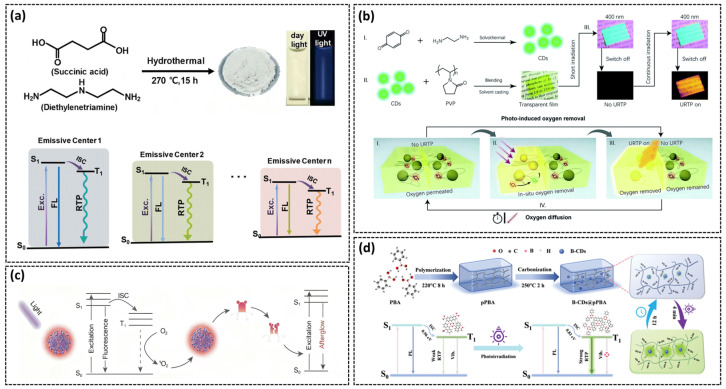
(**a**) Synthesis route of MP-CDs and excitation-dependent FL and RTP emission processes [85]; (**b**) Fabrication process of CD/PVP composite film and demonstration of its dynamic URTP and the processes to achieve a long afterglow [87]; (**c**) Schematic representation of the photooxidation-triggered long-persistent afterglow mechanism in CDs [88]; (**d**) Synthesis strategy of B-CDs@pPBA composites and the mechanism of photo-activation of ultra-long RTP [89].

The afterglow color can be tuned by varying the excitation wavelength, due to the existence of multiple afterglow emission centers (C=O, C=N). Jiang’s research team synthesized CDs rich in N- and O-containing functional groups via a high-temperature hydrothermal treatment of succinic acid and diethylenetriamine [85]. The abundant C=O and C=N moieties not only enhance spin–orbit coupling and promote ISC processes, but also create multiple emission centers with distinct energy gaps (Figure 7a). As the excitation wavelength shifts from 254 to 420 nm, different emission centers are selectively excited, enabling dynamic afterglow color tuning from blue to yellow.

Light irradiation can also modulate afterglow by altering the oxygen content within the system. Molecular oxygen in its ground triplet state (^3^O_2_) can quench phosphorescence through energy transfer with the T state of the phosphor under the excitation of light:

T_1_ (phosphor) + ^3^O_2_ → S_0_ (phosphor) + ^1^O_2_ (singlet oxygen)

This process consumes T-state excitons via an electronic exchange mechanism to quench phosphorescence. Sun’s group demonstrated reversible light-responsive RTP by embedding CDs in a methyl acrylate (PMMA) matrix. The oxygen-permeable PMMA allows ^3^O_2_ to diffuse into the composite, initially quenching the phosphorescence. Under continuous UV irradiation, ^3^O_2_ is converted to ^1^O_2_, reducing its quenching efficiency. When the material is subsequently stored in air, ^3^O_2_ penetrates into the PMMA and the phosphorescence disappears [86]. Liu’s group developed a light-activated ultralong RTP system by embedding luminescent CDs in a PVP matrix [87]. Under light exposure, the CDs generate triplet excitons that convert ambient ^3^O_2_ to ^1^O_2_, creating a localized oxygen-depleted region. This spatially selective hypoxia activates ultralong orange afterglow lasting several hours. After the sample has been exposed to the air for some time, the oxygen in the environment gradually diffuses, and the afterglow disappears (Figure 7b). The difference is that this process is also regulated by temperature. Increasing the temperature can accelerate the penetration of oxygen, thereby shortening the afterglow lifetime.

In some systems, photo-generated reactive oxygen species can chemically modify the CD surface, creating new luminescent centrosomes and thereby altering the afterglow performance of CDs. Lou’s research group developed a photo-oxidation-induced strategy to construct long-lived near-infrared afterglow luminescence CDs, with a afterglow luminescence lifetime up to 5.9 h [88]. Under light excitation, the ground state electrons of CDs transition to the excited state, and then ^1^O_2_ is generated through non-radiative transitions. The reaction not only consumes dissolved oxygen to inhibit the quenching of triplet excitons, but also oxidatively modifies the CD surface to extend the afterglow lifetime (Figure 7c). In the same year, Bi’s team used phenylboronic acid (pPBA) to create a highly reversible photoactivatable phosphorescent composite [89]. Under light exposure, hydroxyl groups in the pPBA matrix are photosensitized by CDs to form oxygen radicals, inducing dynamic cross-linking (e.g., C-O-B, B-O-B). Furthermore, it provides a more rigid matrix environment for B-CD, effectively blocking the non-radiative attenuation channels of the system. Therefore, both the phosphorescence efficiency and the lifespan have been improved. Upon cessation of irradiation, the cross-linking bonds in the system slowly dissociate, restoring the initial flexible matrix and diminishing the phosphorescence intensity (Figure 7d).

### 3.5. Other Responses to the Afterglow

**Figure 8:**
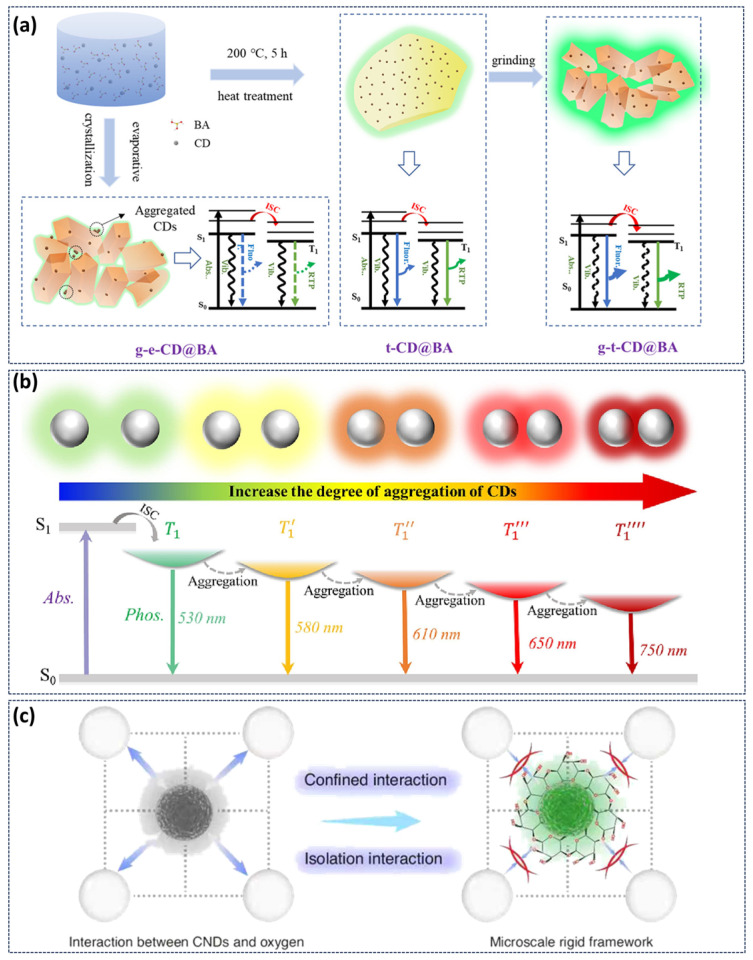
(**a**) Schematic illustration of the phosphorescence mechanism of CDs@BA composite materials under different degrees of aggregation [10]; (**b**) Phosphorescent emission processes and energy level diagrams of composite materials CDs@BA at different aggregation degrees [90]; (**c**) Demonstration of ultrasound-responsive phosphorescent CNDs by microscale rigid framework engineering [91].

In addition to the common stimulus-response types mentioned above, our research group has also developed stimulus-responsive phosphorescent CDs such as force and solvent. In CDs-based crystalline materials, we developed a novel strategy for preparing high-efficiency and strong-emission phosphorescent CDs composites via grinding-induced amorphous-crystal phase transformation of the matrix [10]. High-temperature pretreatment first produces an amorphous BA matrix that facilitates maximum dispersion of CDs without aggregation. Under grinding action, the transient crystallization induced by grinding enables CDs to be in situ and uniformly embedded into the crystal BA matrix (Figure 8a). This approach maximizes CD dispersion within the crystals while significantly increasing their loading capacity. The resulting highly rigid crystalline environment strongly suppresses non-radiative transitions, dramatically enhancing phosphorescence emission of CDs. This method currently holds the record for both the highest phosphorescence efficiency (48%) and highest recorded brightness (590 lumens) among CDs-based materials. In the preparation of multicolor phosphorescent CDs systems by CDs aggregation-induced energy level fission, our research group achieved multicolor phosphorescence through solvent-induced CDs aggregation (Figure 8b), resulting in a reversible phosphorescent color transition from green to red [90]. When placed the sample in a bare environment for more than 30 days, it still exhibited the phosphorescence. Furthermore, samples can be activated repeatedly without a significant loss in the phosphorescence intensity, indicating a high optical stability and signal coherence. Recently, Liu’s team reported ultrasound-responsive phosphorescent CDs using a micro-scale rigid frame engineering strategy (Figure 8c). The local rigidity surrounding the CDs was enhanced by exploiting the ultrasound-triggered self-assembly of cyclodextrin in water, leading to significantly improved phosphorescence performance in aqueous solution with a lifetime extending to 1.25 s [91]. Furthermore, the composites demonstrate excellent reversibility and photostability. The phosphorescence intensity maintained good consistency over five stress–ultrasonic recovery cycles and showed only a slight decrease after continuous UV exposure for 10 min.

## 4. Application

Based on the unique stimulus-responsive characteristics of the afterglow CDs, particularly their sensitivity to temperature and humidity, these materials demonstrate transformative potential in fields including environmental sensing and information security. A series of high-performance sensing and anti-counterfeiting systems has been developed by precisely modulating the afterglow intensity, color and lifespan of CDs.

### 4.1. Sensing

**Figure 9:**
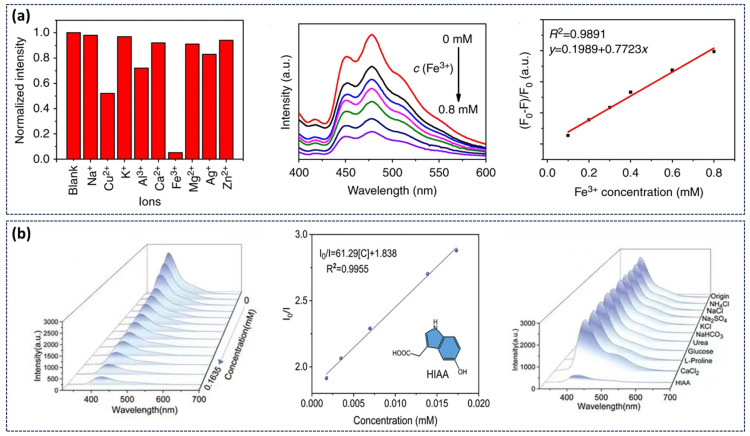
(**a**) Quenching effect of Fe^3+^ ions on the phosphorescence emission of CD-CA composites and the corresponding linear relationship with phosphorescence intensity [57]; (**b**) Phosphorescent emission spectra of m,p-CDs@CA suspension with progressively increasing HIAA concentration and the derived calibration curve for HIAA detection [94].

The application of stimulus-responsive CDs in environmental sensing exploits their distinct luminescence mechanisms and dynamic interactions with external stimuli. Our research group has developed a highly efficient, metal-free RTP material system in aqueous media by constructing a multi-component hydrogen-bonding network involving CDs, CA, and water molecules. The resulting CD-CA suspension can serve as a sensor for detecting metal ions in water [57]. Experimental results demonstrated selective phosphorescence quenching in the presence of Fe^3+^ ions. As Fe^3+^ concentration ions increase, phosphorescence intensity progressively decreased, showing a strong linear correlation (R^2^ = 0.9891) (Figure 9a). Bai’s group synthesized CDs-MgAl-LDHs and explored its application as an oxygen-sensing indicators [93]. The phosphorescence emission intensity exhibited a consistent decrease with increasing oxygen concentration, enabling quantitative oxygen detection. Additionally, Yang’s team employed m,p-CDs@CA suspension as a sensor for detecting 5-hydroxyindole-3-acetic acid (HIAA) in aquatic environments [94]. As shown in Figure 9b, the RTP intensity of m,p-CDs@CA decreased markedly with increasing HIAA concentration, demonstrating its potential for sensitive detection of biomolecules in water.

### 4.2. Information Anti-Counterfeiting Encryption

**Figure 10:**
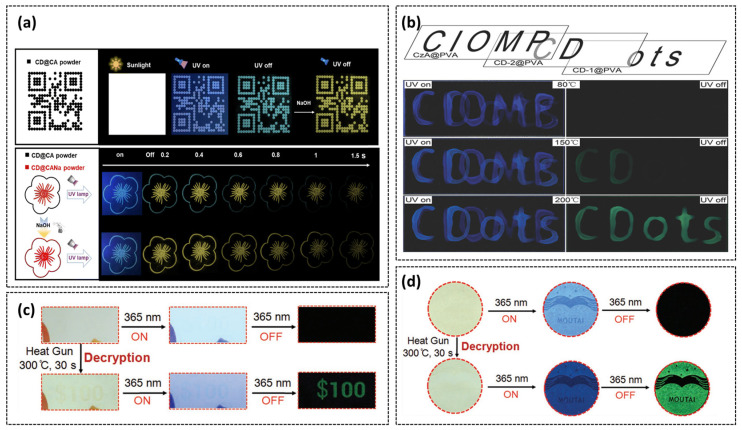
(**a**) CD@CA Acid–Base Responsive Anti-Counterfeiting Application [92]. (**b**) Overlapped fluorescent patterns fabricated with three composites after annealing at 80, 150, and 200 °C [42]; (**c**) Thermal-responsive afterglow authentication of gift certificates using F-CD materials, with verification through phosphorescence emission upon thermal stimulation [34]; (**d**) Anti-counterfeiting application of F-CD based thermal-responsive afterglow technology for Moutai liquor authentication label [34].

The tunable phosphorescence emission and precise environmental responsiveness of stimulus-responsive CDs position them to spearhead the advancement of anti-counterfeiting technology towards higher security and intelligent applications. Given the extreme pH sensitivity of acid–base-responsive CDs’ luminescence, our research group synthesized an alkali-induced selective activation CD@CA [84]. This composite was printed as a model QR code pattern, demonstrating dual-mode display and advanced anti-counterfeiting capabilities. As shown in Figure 10a, the pattern exhibits blue phosphorescence under 254 nm illumination and cyan afterglow thereafter. Upon stimulating the QR pattern with NaOH solution, a yellow afterglow QR code appears following 254 nm illumination. In a floral model printed with CD@CA (petals) and CD@CANa (stamens), the petals show cyan afterglow while the stamens emit yellow afterglow lasting 1.5 s after UV excitation. Alkali spraying transforms the petal afterglow from cyan to yellow, maintaining the 1.5 s duration. Tian’s group developed a temperature-gated encryption system utilizing CD@PVA composites annealed at different temperatures [42]. Multistage fluorescence/phosphorescence responses were achieved to enable multilevel data encryption by thermally treating blue-fluorescent citrate (CzA@PVA), CD-1@PVA and CD-2@PVA. The encryption principle relies on distinct color patterns observed under UV illumination at annealing temperatures of 80 °C, 150 °C, and 200 °C (Figure 10b). Jiang’s group synthesized CEE-type FL self-luminescent polymers F-CDs from ethylenediamine and phosphoric acid, observing URTP phenomena after thermal treatment. Leveraging the unique transition from FL to URTP, further research developed high-security ink for practical application [34]. The ink was directly printed onto $100 gift certificates and Moutai liquor labels, rendering printed information nearly invisible under daylight. Upon applying heat from a hot-air gun to the gift voucher and Moutai anti-counterfeiting label, the presence of the numeric pattern ‘100’ on the voucher or the Moutai trademark becoming visible under phosphorescence indicates authenticity, whereas absence confirms counterfeiting (Figure 10c,d).

## 5. Summary and Outlook

This review systematically summarizes recent research advances in stimulus-responsive afterglow CDs, with particular emphasis on their afterglow mechanism, synthesis strategies, stimulus-responsive types, and the underlying principles governing their afterglow performance. Through heteroatom doping, matrix embedding, or self-protective structure design, CDs can achieve efficient, long-life, and multi-color phosphorescence and DF emission at room temperature. A variety of external stimuli (temperature, humidity, pH, light, force, solvent, etc.) can dynamically control the afterglow intensity, color and lifetime of CDs by regulating the exciton relaxation path, energy level structure or intermolecular interaction. These unique characteristics position stimulus-responsive afterglow CDs as promising candidates for broad applications in chemical sensing, information anti-counterfeiting, and biological imaging. Despite significant progress, several challenges remain to be addressed. Future research should focus on the following aspects.

- Exploration of Novel Stimulus-Responsive Afterglow CDs: Current research primarily focuses on conventional stimuli including temperature, humidity, pH, and light. Future work should expand toward novel stimulus-responsive systems involving electric fields, magnetic fields, biomolecules, and gases. Such developments would significantly enrich the response dimensions of CDs and broaden their application scope in next-generation intelligent responsive materials and devices.
- Development of Multi-Stimulus Response Afterglow CDs: Single-stimulus responses are increasingly insufficient for complex application scenarios. Future efforts should focus on developing CD systems capable of multi-stimulus coordination or orthogonal responses, enabling simultaneous or sequential recognition and feedback of multiple environmental parameters. This advancement will enhance their utility and security in multimodal sensing and encryption platforms.
- Development of Wide Color Gamut Dynamic Color Afterglow CDs: Most current color-changing devices exhibit limited color-shifting ranges, struggling to cover the entire visible spectrum. By precisely tuning the energy level structure of color-changing devices, the types and distribution of luminescent center, and the interactions between the substrate and color-changing devices, it is anticipated to achieve wide color gamut, high-contrast, and reversible dynamic color-shifting behavior spanning ultraviolet to near-infrared wavelengths. This advancement addresses the demands of high-end display applications and anti-counterfeiting requirements.
- Expanding the Application Scope of Stimulus-Responsive Afterglow CDs: Beyond conventional sensing and anti-counterfeiting applications, stimulus-responsive afterglow CDs show considerable potential in biomedical applications (e.g., controlled drug delivery, cellular imaging, and pathological microenvironment response imaging), flexible electronics (e.g., wearable sensors and smart labels), and optical information storage and processing. Future efforts should emphasize interdisciplinary collaboration to accelerate the transition of these functional materials from the laboratory to practical implementation.

## Figures and Tables

**Table 1:** Summary and comparison of CDs materials.

Precursors of CDs	Matrix	Heteroatom Type	Quantum Yield	Lifetime	Ref.
EDTA-2Na	PVA	N, O	—	380 ms	[39]
m-phenylenediamine	PVA	N	—	456 ms	[40]
EDA	SiO_2_	N, O	—	1.62 s	[46]
CA and BA	—	B	23.5%	1.17 s	[51]
m-phenylenediamine	Urea	N	—	2 h	[54]
IPDI	PU	N	11%	8.7 ms	[55]
levofloxacin	—	N, O	4.2%	237 ms, 354 ms	[58]
biuret and urea	—	N	13.9%	0.53 s	[65]
urea and phosphoric acid aqueous solutions	—	N, P	23%	320 ms	[66]
TA	—	O	4.2%	153.6 ms	[67]
1,8-naphthalimide	Al_2_O_3_	N, O	—	60.6–520.6 ms	[68]
CA	BA	O	8.7%	1.6 s	[69]
imide derivatives	PBA	N, O	1.98–21.85%	1.62 s	[70]
diethylenetriamine and phosphoric acid	—	N, P	4.5%	1.48 s	[71]
1,2,4,5-benzenetetramine and (E)-2-methyl-2-butenedioic acid	—	N	0.1%	57.7 ms	[72]
1-butylamine and phosphoric acid aqueous solution	—	N, P	—	1.25 s, 1.74 s	[73]
tetraethylorthosilicate	—	O	7.4%	2.19 s	[74]
glucose and triethylamine trihydrofluoride	—	N, F	3.45%	1045 ms	[75]
1,8-naphthalimide, urea and quinacridone	—	N, O	13.89%, 27.1%, 8.5%	407.2 ms, 431.9 ms, 415.4 ms	[76]

**Table 2:** Summary of Stimulus-Response Mechanisms and Related Research Studies.

Stimulus Type	Response Mechanism	Refs.
Temperature	Molecular thermal motion	[34,46,79,80,81]
pH	Protonation or deprotonation of oxygen-containing groups	[59,82,83]
pH	Deprotonation-induced enhancement of conjugation	[84]
Light	Selective excitation of different emission centers	[85]
Light	Consumption of triplet oxygen via photoexcitation	[86]
Light	Reduce oxygen quenching effects	[87]
Light	Generation of singlet oxygen	[88]
Light	Generation of oxygen free radicals	[89]
Moisture	Formation of hydrogen bond networks	[57]
Solvent	Aggregation-induced energy level splitting	[90]
Grinding	Enhance rigidity and suppress non-radiative transitions	[10]
Ultrasound	Enhance the rigidity and suppress non-radiative transitions	[91]

## Data Availability

No new data were created or analyzed in this study.
